# Long-term Survival after Resection of HER2+ Infiltrating Ductal Carcinoma Metastasis to the Brainstem

**DOI:** 10.7759/cureus.462

**Published:** 2016-01-19

**Authors:** Al-Wala Awad, Hasan A Zaidi, Al-Homam Awad, Robert Spetzler

**Affiliations:** 1 Department of Neurosurgery, Barrow Neurological Institute

**Keywords:** brainstem, breast cancer, ductal carcinoma, her2+, metastasis, trastuzumab

## Abstract

The central nervous system is a common site of metastatic spread from neoplasms in distant organs, including breast, bone, and lung. The decision to surgically treat these metastatic lesions is often challenging, especially in the setting of systemic disease or when eloquent brain regions are involved. Treating metastatic disease in the brainstem can be technically difficult, and in many institutions, considered a contraindication to surgical intervention, given the relatively high risk of new postoperative neurological deficits. Herein, we report a case of metastatic ductal carcinoma of the breast with spread to the pontine-medullary junction that was treated with aggressive surgical resection and chronic hormonal therapy. After surgical excision of the brainstem lesion, the patient remained asymptomatic and was maintained on trastuzumab therapy over a 10-year follow-up period, with no radiographic or clinical evidence of recurrent disease. To our knowledge, this is the first report of a patient treated for a solitary metastasis to the brainstem with long-term survival.

## Introduction

The central nervous system is a common site of metastatic spread from metastases in distant organs, such as the breast, bone, and lungs. When the brainstem is involved, the decision to surgically treat these lesions is often challenging. Herein, we report the case of a patient who had long-term (> 10-year) survival and excellent neurological functional recovery after surgical treatment for metastatic ductal carcinoma at the pontine-medullary junction, followed by chronic hormonal therapy.

## Case presentation

**Figure 1:**
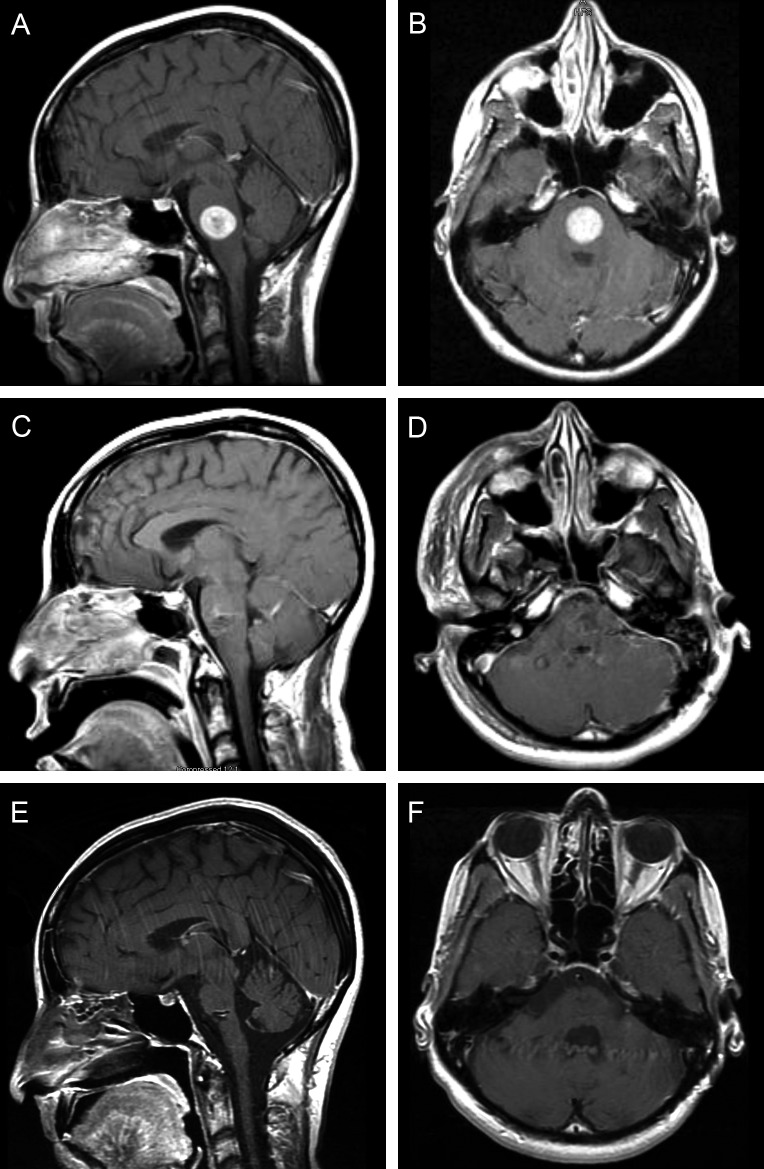
MRI study of the patient's metastatic lesion Initial sagittal (A) and axial (B) magnetic resonance imaging (MRI) scans with contrast of the head demonstrating a well-circumscribed enhancing lesion with surrounding edema in the pons. Postoperative MRI (C-D) with contrast demonstrating a gross total resection of the solitary lesion with no evidence of residual disease. MRI with contrast at 10 years (E-F) after the original craniotomy showed no signs of recurrent disease. *Used with permission from Barrow Neurological Institute, Phoenix, Arizona.*

A 41-year-old female patient with a one-year history of infiltrative ductal carcinoma presented to our clinic complaining of 10 days of worsening headache, tinnitus, ataxia, and paresthesia in the right arm. On neurological examination, she was found to have bilateral dysmetria, hyperreflexia of both upper and lower extremities, and a wide-based gait. The cranial nerves of the patient were otherwise intact, and she had normal 5/5 motor strength throughout. Because of the recent onset of her symptoms, a magnetic resonance imaging (MRI) study was performed, which demonstrated a 2.0 × 2.1 cm, well-circumscribed, contrast-enhancing lesion centered at the pontine-medullary junction (Figure 1A, 1B).

The patient’s medical history included a left breast mass found incidentally on routine mammography one year prior to the current presentation. At that time, the patient underwent a radical lymphadenectomy, which showed 14 of 23 positive nodes of a Stage IIIC human epidermal growth factor receptor 2–positive (HER2+), estrogen/progesterone receptor-negative, infiltrative ductal carcinoma. She underwent six months of chemotherapy (doxorubicin and paclitaxel) and then completed one year of hormonal therapy with the HER2+ antagonist, trastuzumab. During this period, there was no indication of recurrent disease until the aforementioned presentation.

After discussing potential risks and morbidity associated with brainstem surgical resection, as well as the alternatives (e.g., radiotherapy and chemotherapy without surgery), the patient was adamant about pursuing aggressive surgical resection. Informed patient consent was obtained. After registration with the StealthStation surgical neuronavigation system (Medtronic, plc, Dublin, Ireland) using contours of the scalp and confirming accuracy using known anatomical landmarks, a standard left-sided retrosigmoid craniotomy was performed. A wide arachnoid dissection was performed, and brain relaxation was achieved by opening the basal cisterns and releasing cerebrospinal fluid. This obviated the need for retractors or a lumbar drain. An optimal trajectory was chosen to the center of the lesion using the previously described two-point method and the neuronavigation guidance system [1]. Given the anatomical configuration of this particular mass deep within the pons, the middle cerebellar peduncle was entered to access the lesion, a procedure that is relatively well-tolerated in most patients. After cauterization of a small portion of the pia with bipolar electrocautery, the middle cerebellar peduncle was entered using a combination of bipolars and suction. A partially hemorrhagic, well-circumscribed lesion was encountered deep within the pons. A combination of cupped dissectors and toothed forceps allowed the specimen to be circumferentially dissected from the surrounding brainstem, with an excellent plane, and to be removed as a single mass. The microscope was angled, and lighted suction was used to inspect the resection cavity for residual disease. After documentation of minimal bleeding within the resection cavity, the dura and skin were closed in a watertight fashion and the patient was transferred to the intensive care unit for overnight monitoring.

Postoperatively, the patient suffered transient 6th and 7th nerve palsy on the left, right-sided hemiparesis, and continued right-sided paresthesia. On postoperative day 5, she was discharged to a rehabilitation facility. With physical therapy, her neurological deficits rapidly subsided. At her two-month follow-up clinic visit, she had normal facial symmetry, no diplopia with left lateral gaze, normal gait, and normal strength in all extremities. However, she continued to experience intermittent paresthesia that was not debilitating. Postoperative MRI demonstrated a gross total resection (Figure 1C, 1D), with only minor areas of enhancement that were interpreted as postoperative hyperemia. The patient was monitored with serial MRIs every three months for the first year, every six months for the second year, and annually thereafter. Serial imaging over the following decade demonstrated no further enhancement or evidence of residual tumor. Pathologic specimen analysis confirmed a diagnosis of metastatic ductal carcinoma with a receptor profile identical to that of the original breast lesion. The patient elected to not undergo additional radiotherapy or chemotherapy after the resection of her metastatic brainstem lesion; instead, she opted to continue on chronic trastuzumab therapy and was followed for 10 years. Remarkably, throughout this time, she remained neurologically stable compared to her postoperative state, with no radiographic (Figure 1E, 1F) or clinical signs of tumor recurrence. To date, the patient remains on trastuzumab therapy.

## Discussion

Up to 40% of patients with cancer develop brain metastases, with breast cancer accounting for 5-15% of cases [2-3]. Rates of metastasis and survival depend heavily on tumor subtypes. Patients with HER2+ subtypes have higher rates of metastasis and, thus, have lower rates of survival than patients with the less severe luminal A and B subtypes [4]. However, as treatment regimens with trastuzumab have become more standardized, five-year survival rates have increased to 92% [5].

Surgical treatment followed by radiotherapy for single metastatic lesions to the brain can significantly improve overall survival compared to radiosurgery alone [6-7]. In lesions located in the brainstem, both the surgical extent of resection and radiotherapy can be limited by the eloquence of the surrounding brain tissue. Gamma Knife and fractionated whole brain radiotherapy to brainstem lesions have been shown to increase survival, but carry a high risk of delayed neurological morbidity secondary to scatter of radiation into the surrounding normal brain tissue [7-8]. Post-treatment radiation necrosis and edema can also result in significant neurologic deficits [9-10]. In this case, the patient was adamantly against radiotherapy. Given the well-encapsulated nature of her lesion, complete surgical excision was possible with only transient postoperative neurological deficits. However, if the lesion had been too infiltrating into the surrounding parenchyma or vascular in nature, surgery would have been contraindicated.

Immediately after the original diagnosis of breast cancer and radical lymphadenectomy, the patient completed a six-month course of combination chemotherapy (doxorubicin and paclitaxel) and began trastuzumab therapy. Within one year, she was found to have a single metastatic lesion to the pontine-medullary junction, for which she underwent gross total resection and was maintained on trastuzumab therapy. There was no evidence of local or distant metastasis over a 10-year follow-up period. We found only one other report in our review of the medical literature that documented such long-term use of trastuzumab. In Witzel, et al.’s [11] report on the long-term survival of 268 patients with HER2+ breast cancer, only six patients had metastasis to the central nervous system. In this large cohort of patients, the median time to progression was 4.5 years while on continuous trastuzumab therapy, and three patients had documented progression-free survival of 10 years. The authors of the study did not indicate whether any of these survivors had central nervous system metastasis. Only 20% of the patients in this study were treated with trastuzumab therapy alone; the remainder initially received a combination of chemotherapy and endocrine agent (tamoxifen) [11], as did our patient. Importantly, a determinate of a reduced progression-free survival was the interruption of trastuzumab therapy [11]. Interestingly, the Herceptin Adjuvant (HERA) trial demonstrated that two years of trastuzumab therapy did not provide a greater survival advantage than one year [12]. In our patient, the decision to treat continuously with trastuzumab was based on several factors, mainly, the lack of established guidelines at the time of treatment and the willingness of the patient to continue long-term treatment given the high rate of relapse associated with HER2+ infiltrative ductal carcinoma [4].

## Conclusions

Brain metastases are common in patients with breast cancer. The extent of treatment is challenging and unique to each patient’s disease status and lesion location. Infiltrative ductal carcinoma is a common breast cancer that has seen improved prognosis in patients with HER2+ receptor subtypes, including patients with metastatic spread to the brain. Even in the setting of brainstem metastasis, aggressive surgical resection followed by adjuvant trastuzumab therapy can provide meaningful recovery and long-term survival.
